# Key factors influencing canine heartworm, *Dirofilaria immitis*, in the United States

**DOI:** 10.1186/1756-3305-5-245

**Published:** 2012-10-30

**Authors:** Heidi E Brown, Laura C Harrington, Phillip E Kaufman, Tanja McKay, Dwight D Bowman, C Thomas Nelson, Dongmei Wang, Robert Lund

**Affiliations:** 1 School of Geography and Development, University of Arizona, Tucson, AZ, 85721, USA; 2 Department of Entomology, Cornell University, Ithaca, NY, 14853, USA; 3 Entomology and Nematology Department, University of Florida, Gainesville, FL, 32611, USA; 4 Department of Biological Sciences, Arkansas State University, State University, AR, 72467, USA; 5 Department of Microbiology and Immunology, College of Veterinary Medicine, Cornell University, Ithaca, NY, 14853, USA; 6 Animal Medical Center, Anniston, AL, 36201, USA; 7 Department of Mathematical Sciences, Clemson University, Clemson, SC, 29634-0975, USA

**Keywords:** Canine heartworm, *Dirofilaria immitis*, Mosquito vectors, Spatial prevalence

## Abstract

An examination of the Companion Animal Parasite Council’s (CAPC) canine heartworm data to clarify the spatial prevalence of heartworm in the United States. Factors thought to influence the spatial risk of disease, as identified in a recent CAPC workshop, are discussed.

## Background

The Companion Animal Parasite Council (CAPC) collected results of 4,769,403 canine heartworm tests during the 2011 calendar year from various commercial testing laboratories in the United States (US), and of this national sampling of dogs, 56,612 (1.187%) were positive for heartworm antigen, indicative of an active *D. immitis* infection [1]. This rich data set can be used to infer the national prevalence of heartworm disease and/or verify the accuracy of predictions made from forecasting models. Herein, we: 1) present a map of positive heartworm test prevalence, thereby upgrading existing knowledge; and 2) construct a list of factors that will be used to explain the observed rates of heartworm positive tests over the coming years of similar data collection.

This work stems from a meeting held in Atlanta, GA, on June 9–10, 2012, during which vector ecologists, entomologists, and biologists worked with a team of statisticians to identify risk factors which could be useful for the development of spatial risk mapping for important vector-borne canine diseases for which CAPC had access to collected data with the overarching objective being to identify the most important factors influencing Lyme, ehrlichiosis, anaplasmosis, and heartworm infection rates in the US canine population [1]. The focus of this paper is on one of these data sets, i.e., the data relative to canine heartworm (*Dirofilaria immitis*) infection.

Heartworm infections are a significant health risk to dogs as even light infections are capable of producing profound pulmonary vascular and parenchymal disease. Despite improved diagnostic methods, effective preventives and increasing awareness among veterinary professionals and pet owners, cases of heartworm infection continue to be diagnosed in high numbers and are becoming more prevalent in areas previously considered to be at a low risk [2]. A survey of veterinary clinics in 2005 reported that over 250,000 dogs tested positive for heartworms during the 2004 calendar year [3]. When one considers a 48% response rate to the survey and the fact that only 30% of the dog population in the US was tested, the actual numbers of dogs infected are much higher, probably in the 1 to 1.5 million range.

The test used for antigen detection in dogs has a high sensitivity (84%, n= 175/208) and high specificity (97%, n= 30/31) [4]. While concerns about false positives in areas of low prevalence exist, comparisons with known prevalence rates and studies that have examined dogs for the presence of microfilariae indicate that any overestimation of the infection rates due to false positives is not large. A survey for microfilarial presence rather than antigenemia conducted in Colorado found an overall prevalence of microfilarial positive dogs in 1990 to be 0.77% for 7,818 dogs tested [5]; the 2008 data showed a prevalence in Colorado of 0.4% [2]. In 1981–1982, a survey of 541 dogs in 12 cities and four counties in Northern California found that 31 (5.7%) were positive for heartworm microfilariae [6]; the prevalence rates in these counties by microfilariae were similar to results from the antigen survey [2].

**Figure 1:**
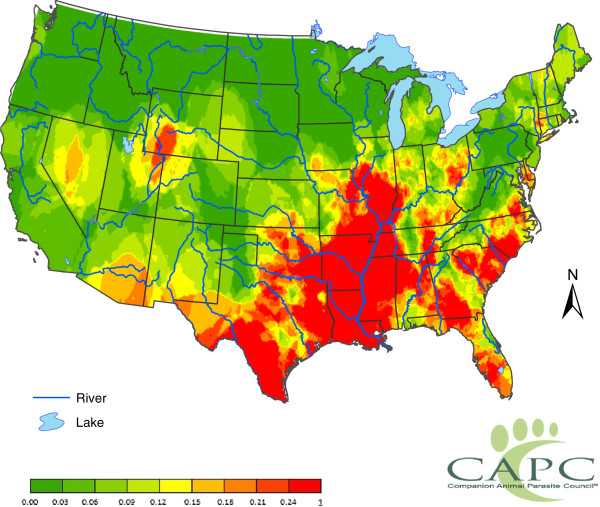
**Spatially smoothed proportions of positive canine heartworm**-**antigen tests recorded by US veterinarians in 2011.** The figure summarizes 4,769,403 tests performed by veterinarians for circulating heartworm antigen in the US in 2011; of these tests, 56,612 tests (1.187%) were positive. The population studied is those dogs that are seen by a veterinarian and are tested; about 5% of owned dogs in the US. The map displays probabilities of a positive test after smoothing by the head-banging algorithm (see text for details). The figure is made by assigning the smoothed proportions to nine color-coded categories [0.00,0.03], (0.03,0.06], . . .,(0.21,0.24], and (0.24, 1.00]. The colors range from dark green for the lower proportions to bright red for higher proportions.

An example of the information that the CAPC heartworm database contains is represented by a map displaying spatially smoothed heartworm positive tests for the 2011 calendar year (Figure 1). The graphic shows that heartworm is most prominent in the lower Mississippi River Valley and nearly absent in Northern Montana. This map was generated using annualized data --- no seasonal features were considered. The results represent the prevalence of positive tests for circulating heartworm antigen over a full year amongst a population of dogs that are visiting a veterinarian and receiving care that includes at the least a test for heartworm infection. The proportion of positive heartworm tests was computed for each county in the contiguous 48 states. Proportions are analyzed in preference to total positive test counts as the number of positive tests can be influenced by local testing practices. These proportions were then spatially smoothed via a procedure known as the head-banging algorithm and grouped into nine probabilistic categories. The methods account for the varying number of tests made in different counties; for example, ten positive tests in a sample of size one hundred statistically suggests more of a problem than one positive test in ten, although the sample infection proportions are the same. The map will be updated in future years as additional data become available. While this map quantifies the baseline heartworm infection rates for US counties, it is desirable to understand what factors explain heartworm risk. Our immediate goal was to assemble a list of factors, which are available and quantifiable, that may influence canine heartworm infection rates.

At the June 2012 CAPC meeting, a team of experts provided up to ten measurable factors, ranked in order of importance that are most likely to affect heartworm prevalence and can be used for spatial risk mapping. It was understood that some of the factors might prove to be of no value, that some could be of significant value, that there might be interactions between factors, and that some important factors could be omitted or non-measurable. Because heartworm risk varies both spatially and temporally, the team included factors which could predict both baseline prevalence rates and inter-annual variations. Before leaving the meeting, a ranked list was generated (Table 1).

**Table 1:** Ranked factors initially identified by the working group

	
1	Vector presence (+)
2	HDU (calculated from weather with a 14°C threshold) (±)
3	Lagged weather (7 months to 24 months prior to diagnosis)
	a. Precipitation, temperature, and or relative humidity (±)
	b. Moisture Index (Precipitation and Evaporation) (±)
4	Presence of coyote and feral dog populations – categorical data (+)
5	Human population density (+)
6	Land cover (Dominant type or Percentage classification) (±)
7	Cropland (Dominant type or Percentage classification) (±)
8	Social economic status (±)
	a. Household income (-)
	b. Education (-)
	c. Foreclosure rates (+)
9	Irrigation (±)

## Factors and their selection rationale

The factors selected by the heartworm working group are listed and discussed here, grouped by vector, parasite, and host factors. Some factors, such as temperature, are important to multiple factor groups (vector and parasite). Two criteria in selecting factors were that 1) the factor can be easily measured and 2) data for it are available.

### Vector factors

Dog heartworm is a mosquito-transmitted disease with a cosmopolitan distribution [7,8]. The mosquito becomes infected when it ingests microfilaria during the act of blood feeding on an infectious host. To be a competent vector of *D. immitis*, mosquitoes must be able to support nematode development into the infective stage (L3), and the infective larvae must be able to migrate to the proboscis of the mosquito [9]. Over 60 species of mosquitoes are capable of supporting the development of L3 *D. immitis*[7]. Summaries of mosquito species naturally infected with *D. immitis* in 19 states have appeared in previous publications [10-12]. In the US, multiple studies on potential heartworm vectors have been conducted; however, most studies were restricted to certain locations. Without known vector distribution data from the majority of states, estimates are needed to identify areas where each vector occurs and to what abundance. This distributional data can then be used to identify areas with greater risk for heartworm transmission. With a number of mosquito species likely playing different roles across different regions of the US [9] and taking into account mosquito vector competence data either obtained naturally and/or experimentally, nine species were identified as major potential vectors for national forecast modeling. These species are *Aedes aegypti, Aedes albopictus*, *Aedes canadensis*, *Aedes sierrensis, Aedes trivitattus, Aedes vexans*, *Anopheles punctipennis*, *Anopheles quadrimaculatus*, and *Culex quinquefasciatus*.

For most of the nine mosquito species chosen, published maps illustrating their geographical home ranges within North America exist [12]. As many of the mosquito species have varying frequency distributions within even small ranges, the historical literature can serve as a starting point for fine-tuning the representation of their ranges. For species, such as *Ae. albopictus* and *Ae. aegypti,* where competition between the species has led to changes in their historical ranges [13,14], new geographical distribution data can be made available as it is accumulated.

Additional vector maps can be generated using surveillance data and habitat modeling, but quality surveillance data are notoriously difficult to acquire. Consequently, there is a need to use surrogate factors. Possible surrogates for actual presence/absence or abundance maps of these mosquito species include: 1) vegetation indices (derived from satellite imagery or classified land cover maps); 2) urbanization as it relates to rural, suburban, and urban landscapes; and 3) meteorological data to capture inter-annual variability. As new mosquito distribution maps are generated, they will ideally be examined by entomologists to verify that they represent current species ranges.

#### Vegetation indices

A synopsis of various mosquito species that breed in different habitats can be made available and overlaid on land cover, cropland, and irrigation maps of the US. For example, previous studies that have focused on mosquitoes associated with forested environments (*Ae. sierrensis* and *An. punctipennis*) [15], rice production (*An. quadrimaculatus*) [16], and irrigation could provide data for map generation. These data can be included both as dominant type of vegetation/cropland or as a percentage of each type by county.

#### Rural, suburban, and urban landscapes

Certain mosquito species are more prevalent in urban environments due to differences in their preference for particular oviposition sites. Mosquito species that breed in artificial containers tend to be more urbanized, such as *Cx. quinquefasciatus* and *Ae. aegypti*. The spatial and temporal distribution of *Cx. quinquefasciatus* in residential areas [17] and underground storm drains has been well documented [18,19], and this type of information may be useful for modeling. Human population data from the US Census Bureau and land cover classified imagery with urban, rural, suburban land use classes may be helpful inputs for the model to capture measures of urbanization.

#### Meteorological data

Mosquito development rates are temperature- dependent and completion of the immature stages is dependent on water availability. In a forecasting model, weather (daily and monthly, and at various time lags) is expected to influence intra-annual fluctuations. In a static spatial model, climate, rather than weather, may better govern the likelihood a vector can complete its lifecycle. In an effort to incorporate these influences, we recommend including temperature (minimum, maximum, mean, and daily variability) and moisture index (calculated based on precipitation and evaporation rates) [20]. The moisture index *M*(*t*) at day *t* over the current and previous *L* days is

$$M\left(t\right)=\Sigma_{i=t-L}^{t}P\left(i\right)-D\left(i\right)\text{,}$$

where *P*(*i*) and E(*i*) are the precipitation and evaporation from day *i*, respectively. Moisture indices may be more meaningful than rainfall because they take into account the moisture deficit prior to rainfall and estimate the net moisture in terms of standing water available to egg-laying mosquitoes.

Related indirect factors that could plausibly influence heartworms include: 1) relative humidity, 2) elevation, and 3) daily circadian-related events in mosquito behavior. Relative humidity influences vector behavior and survival. Elevation may serve as a surrogate of the climate expected at a given location. Circadian activity is important to some vector species’ behavior [21,22].

### Parasite factors

Several issues were identified relative to the parasite and its development within the vector and host. Heartworm Development Units (HDUs) as they relate to nematode development in mosquitoes and the lag period between infection and detection in the data on the current antigen detection maps were considered other important factors.

The *D. immitis* lifecycle consists of several developmental stages in both the mosquito vector and vertebrate hosts [9,23-25]. Heartworm transmission is driven by the ambient temperatures experienced by their mosquito vectors. As a consequence, ambient air temperature is used to predict the timing of dog heartworm vector competence [26,27]. The heat requirement for heartworms to complete incubation to the infective stage can be expressed in development degree days, or HDUs. A simple way to calculate HDUs using maximum and minimum air temperatures is to subtract the heartworm development threshold (14°C) from the average daily temperature [9,26]. Accumulated HDUs are summed from a determined starting day, similar to the moisture index equation above. If hourly temperatures are utilized, the summation of the difference in hourly temperature from 14°C can be divided by 24. The number of accumulated degree days required for development to infective parasite stage is 130 [27], although this value may vary by strain of the parasite. *D. immitis* larval survival and development within a mosquito is consequently dependent on temperature and its fluctuations --- larval heartworms are capable of slowing and recommencing development in a temperature-dependent fashion. In addition, *D. immitis* larval survival is dependent on survival of its invertebrate vector as the infected mosquito must survive the parasite burden and ingest another blood meal from a dog in order to transmit the infective larval stages. Taken together, these findings suggest that predicting future temperature variation and accumulated thermal units may be key in predicting heartworm.

The *D. immitis* antigen does not appear in the blood of heartworm infected dogs until 6 to 9 months after infection [9,23,27,28]. Therefore, a positive test for heartworm often indicates an infection acquired sometime during the previous year. The CAPC dataset does not include information on whether positive cases are regular or new patients. Therefore, all cases are viewed to represent infections acquired more than 6 months previously. As these data are used to develop forecasting models, it will be critical that factors are considered over an appropriate history. We recommend examining factors between 7 months and 2 years prior to the current date.

### Host factors

Heartworm hosts, principally the domestic dog, *Canis familiaris*, but wild canids also maintain the parasite and infect mosquitoes [11]. Effective heartworm prevention is available for domestic dogs, but compliance is insufficient to control the disease (only 74%-79% of dogs visiting the veterinary teaching hospital at the University of Pennsylvania [VHUP] from January 1999 through December 2006 were being given preventive at any given time of year) [29].

Measures of the size and infection status of these susceptible and at-risk populations will be important to estimating the spatial risk of disease. Although dogs evacuated from the Gulf coast states following the 2005 hurricanes presented as 48.8% dirofilariasis-positive [30], the percentage positive rate among the general population of owned dogs is expected to be much less. Ideally, data would be available on the susceptible population of companion animals, coyotes, and feral dogs. However, such data are likely difficult to acquire. Surrogates for assessing the susceptible population include estimates of feral /wild populations, sales data on HW prevention for companion animals, and socioeconomic factors.

#### Unprotected reservoirs

Feral dogs and coyotes are perhaps the most significant heartworm reservoirs in North America as these competent reservoir populations are not covered through a prophylactic program. Indeed, many infections in non-protected domestic dogs (or dogs receiving inadequate protection) are probably a major source of infection via mosquitoes for other domestic dogs. These populations support parasite development and routinely have circulating microfilariae infective to mosquitoes [31-33]. Unfortunately, accurate data on their population size and the prevalence of heartworm within a given population is difficult to obtain, however, the working group considered this information to be valuable. As such, obtaining population size data for coyotes from wildlife authorities and on stray dogs from animal control agencies could be achieved through telephone inquiries. Methods have been developed that provide for coyote population estimates and their relative reliability [34].

Background heartworm prevalence in reservoir populations could be estimated from published coyote survey results, as well data from dogs in shelters. Many reports on coyote and other wild canid species’ *Dirofilaria* infections have been published with report rates as high as 71% [35]; however, most surveys occurred in the early 1980’s [36]. More recent surveys conducted in Oklahoma/Texas, Illinois, Florida and California have reported 6.5, 16, 40 and 42-44% of coyotes being positive [36-40]. In contrast, studies in Arizona and eastern Washington state reported no coyote infections [41,42], while a separate recent survey of Arizona wild canids (feral dogs and coyotes) reported that 14% were positive for heartworm [43]. Such divergent results emphasize the care needed to utilize a sentinel survey of feral canines.

#### Regional data on the percentage of dogs on prevention or doses of product sold

There are two populations of canids in any heartworm infected area: canids on preventive therapy and canids that are not. Many of the dogs being tested are likely from the protected population. As the protection status of each tested dog is not available in the CAPC data, surrogate factors might prove useful. For example, data on the total dog-equivalent doses sold in a given area could serve as an indicator for number of animals in an area on heartworm prevention. A few studies have attempted to estimate the percentage of dogs on preventatives through owner surveys [29], but it is difficult to generalize nationally from geographically localized small-scale surveys. Such data, therefore, are not necessarily a good representation of any certain clinic’s patient demographics. Other sources of potential heartworm preventative use would involve pharmaceutical companies’ sales data.

#### Social economic status

In a study conducted of patients presented at a Pennsylvania teaching hospital, patient age, owner household income, and being neutered were factors that were associated with an increased likelihood of heartworm preventative compliance [29]. At the highest income levels in the study, compliance began to drop, sometimes considerably, thereby adding a level of caution when using a linear association with socioeconomic status. In the case of the dogs shipped out of Louisiana after Hurricane Katrina, intact dogs were 1.6 times more likely to have dirofilariasis than neutered dogs [30].

A relationship between pet relinquishment and foreclosures has been established in California [44]. Areas with concentrated foreclosures had greater concentrations of pet relinquishments. Furthermore, it was reported that residents in low-valued homes are more likely to have un-neutered dogs and were more likely to relinquish these animals, and that neuter status and relinquishment shifted as home values increased [44]. Taken with the lower compliance by owners of reported unneutered dogs [29], these factors may be valuable in the model development.

### Alternative factors

In addition to the factors discussed above, a list of covariates, secondary in importance to the ones above, were discussed. These secondary covariates include:

#### Latitude

While the effect of latitude may depend on the location, latitude could serve as a surrogate for general weather, which influences the presence and abundance of mosquito species [45]. However, because the more nuanced and finer resolution meteorological data are available for use as potential factors, latitude may be duplicative to these other factors.

#### Geographic mobility or migration behavior of humans (clients) within a given area

As pet owners with unprotected pets move between high and low transmission regions, they may be facilitating heartworm spread to new areas (see host factors above). Bringing infectious or susceptible dogs into new areas may influence the spread and inter-annual incidence of heartworm. Geographic mobility data may be important in identifying heartworm outbreaks. This said, the factor may be captured in demographic data already described above, and is hence considered secondary.

#### Pollen count

In regions where precipitation is low (the Southwest for example) or where irrigation is common, pollen may serve as a surrogate for vector habitat. Pollen counts may be a better measure in regions where precipitation underestimates the availability of immature habitat. However, with the inclusion of crop types, forestation coverage, and meteorological data, pollen counts seem less important.

### Factors with insufficient data for inclusion

A number of factors considered important seemed unlikely to be sufficiently quantified for use in modeling. These factors can be divided into two groups: those important to vector and parasite development and those important to host susceptibility. Some of the vector and parasite factors excluded were: vector infection rates, detailed reservoir infection rates, vector abundance and flight range, vector competence (vector efficiency index), vector survival, temperature-dependent development rates of vectors (under natural fluctuating temperature regimes), and variations in heartworm development thresholds. These factors are important for accurate forecasting models because they dictate the rates at which vectors emerge in the population and become infectious. However, due to lack of data availability, we excluded them from the model factors list.

The number of susceptible hosts in a population is also important. Direct measures of susceptible populations are not available. Potential surrogates for these data are presented above.

## Data limitations

Concern was expressed that not all of the above factors are easily obtainable or recorded to a fine geographical resolution, which may bias the model predictions. County-level data might be misleading in some locales --- a single county can encompass multiple climate zones in western parts of the US. County size and homogeneity of the above factors (e.g., human population density, crop types, and climates) will vary geographically. There also was concern over what the data truly represent. Differences in testing procedures, which may vary geographically, may influence the model predictions. In addition, testing rates are likely to not be evenly distributed across populations such that certain populations, which may have a considerable role in disease transmission, may not be adequately captured. Aspects of these limitations can be addressed statistically; others will be discussed with respect to interpretation of the model output.

## Data sources

Data are available for many of the key factors. Geographic data are readily available from the US Geologic Survey Land Cover Institute and the National Atlas of the US [46,47]. Additional land cover data can be acquired from the US Department of Agriculture Economic Research Service and the University of Nebraska – Lincoln National Drought Mitigation Center [48,49]. Demographic data are available from the US Census Bureau [50].

## Methods

A spatial smoothing procedure based on the head-banging algorithm method described by Hansen is used to create a first baseline map (Figure 1) [51]. The head-banging algorithm is particularly useful in describing data with high local variations as it is median polished (not easily influenced by outliers). This algorithm is named from a child’s game where a face is pressed against a board of pins protruding at various lengths, leaving a general impression of the child’s face while smoothing away any excessively-varying local features that are more attributable to random chance. We prefer head-banging to classical Kriging smoothing techniques since the latter could be unduly influenced by a few counties with high heartworm prevalence.

A complication is that varying numbers of dogs were tested in distinct counties. To handle this aspect, the county data is converted to a common basis via standard normal Z-scores. Here, *p*(*s*) is the probability that a single tested dog is heartworm positive at location (county), *s*. If *N*(*s*) tests were conducted in this county, *k*(*s*) of which are positive, then *p*(*s*) is simply estimated as *k*(*s*)/*N*(*s*). The standard normal Z-score is this estimated probability divided by its standard error:

$$Z\left(s\right)=\frac{k\left(s\right)/N\left(s\right)}{{\left\{\frac{k\left(s\right)/N\left(s\right)\left(1-k\left(s\right)/N\left(s\right)\right)}{N\left(s\right)}\right\}}^{\left\{\frac{1}{2}\right\}}}\text{.}$$

Counties where no tests are performed do not influence the analysis. Our conventions take *Z*(*s*)=10,000 when all tests in the county are positive, and *Z*(*s*)=0 when all tests in the county are negative (these somewhat arbitrary conventions are needed to prevent division by zero).

After Z-scores are computed for each county, the head-banging algorithm is applied to spatially smooth them. From the smoothed Z-scores and the county-by-county values of *N*, one can then convert back to a smoothed probability, representing the probability of a positive test in each county. Figure 1 is a geographic display of these smoothed probabilities using 20 nearest neighbors (these are viewed as adjacent counties).

The smoothed county, *s*, estimate of *p*(*s*), denoted by p*(*s*), will be key in our factor identification task. Specifically, after the smoothed probability estimates are computed, we will consider logistic regression models of the form

$$\text{Logit}(p^{*}(s))=\mu +\beta_{1}f_{1}\left(s\right)+...+\beta_{L}f_{L}\left(s\right)+\alpha \left(s\right)\text{,}$$

where *L* is the number of factors *β*_1_ . . . , *β*_L_ are regression coefficients, *f*_1_(*s*),…,*f*_*L*_(*s*) are values of the observed regression factors for county *s*, *μ* is an overall location parameter, and *α*(*s*) is zero mean random error for county, *s*. Here, logit is defined for values in [0,1] (that is, probabilities) via

$$\text{Logit}\left(p^{*}\left(s\right)\right)=\log\left(p^{*}\left(s\right)\right)-\log\left(1-p^{*}\left(s\right)\right)\text{.}$$

Factor *j* is significant in the prediction of a positive heartworm test if *β*_*j*_≠0. Standard forward and backwards regression model factor selection routines can be used to determine which of the *L* factors are significant and how significant is each factor. The interested reader is referred to Casella and Berger (2002) for further elaboration [52].

Forecasts of future prevalence can be obtained from the above logistic regression model as various predictors and data from future years are considered. By inverting the inverting the logit transform of the regression model utilizing forecasted predictor factors, can be an estimated value of p*(*s*) is obtained. For example, if annual temperature is an important factor, one could use historical temperature data to forecast next year’s annual average temperature. This forecasted factor is then used in the regression equation along with its accompanying estimated value of *β*. Right now, any such forecasts are annual in nature as no seasonality has been considered. However, after a few years of data are collected, it may be possible to quantify seasonal effects and make monthly forecasts. The CAPC data is updated monthly.

## Discussion

Identifying factors involved in heartworm disease is not a new endeavor. Initial work in this area occurred in Canada and the US [26,27], and this has since led to studies on the prediction of the different transmission seasons in Europe [53], the United Kingdom [54] and Argentina [55]. These studies predict the beginning and end of seasonal transmission, and some consider the impact of climate change [56]. Utilizing the rich CAPC data base we have access to, we expect to obtain a clearer understanding of canine heartworm transmission in the US. Our working group identified several factors that could be used in model development. These factors, combined with the modeling approaches outlined above, will be fitted to the comprehensive CAPC data set in the future to generate detailed estimation of canine heartworm risk at a county-by-county resolution in the US.

## Abbreviations

*Ae*: *Aedes*; *An*: *Anopheles*; CAPC: Companion Animal Parasite Council; *Cx*: *Culex*; *D*: *Dirofilaria*; HDU: Heartworm Degree Unit; VHUP: Veterinary Hospital at the University of Pennsylvania.

## Competing interests

The authors have no competing interests relative to the work presented in this report.

## Authors’ contributions

DW and RL were responsible for the statistical presentation and the production of Figure 1. HEB, LCH, PK, and TM were the group participants who identified the risk factors, built the framework for their inclusion into the manuscript, and provided the rationale in the paper for their inclusion. DDB and CTN were responsible for generating the initial draft document from the minutes of the meeting for circulation to the group, the compilation of the later drafts of the document, incorporation of all comments, and assistance with formatting for final submission. All authors read and approved the final version of the manuscript.
